# Possible Mechanisms Involved in the Cooccurrence of Oral Lichen Planus and Hashimoto's Thyroiditis

**DOI:** 10.1155/2020/6309238

**Published:** 2020-02-04

**Authors:** Peiyao Wu, Shuhan Luo, Tao Zhou, Rui Wang, Xuemei Qiu, Peiyang Yuan, Yuqing Yang, Qi Han, Lu Jiang

**Affiliations:** State Key Laboratory of Oral Diseases, National Clinical Research Center for Oral Diseases, Department of Oral Medicine, West China Hospital of Stomatology, Sichuan University, Chengdu, Sichuan, China

## Abstract

Oral lichen planus (OLP) is a chronic inflammatory oral mucosal disorder mediated by T cells, with a multifactorial etiology. Hashimoto's thyroiditis (HT) is a common autoimmune disease characterized by hypothyroidism. Although many clinical studies conducted over the past several decades have reported the cooccurrence of OLP and HT, the underlying mechanism remains unclear. This review summarizes potential mechanisms that might be involved in the cooccurrence of OLP and HT. We find that OLP and HT share a common or overlapping pathogenesis in terms of immune, heredity, environmental, and hormonal factors, which might cause cooccurrence. Furthermore, considering the latency of HT, a routine screen for thyroid diseases, particularly HT, is suggested for confirmed OLP patients.

## 1. Introduction

Oral lichen planus (OLP) is a relatively common chronic inflammatory disease of the oral mucosa affecting 0.5–2% of the population; middle-aged and elderly female populations are more commonly affected [1]. Although OLP etiopathology remains unknown, it is believed that immune dysregulation [2], psychological factors [3], and genetics [4] play crucial roles. Hashimoto's thyroiditis (HT) is characterized by the aggregation of autoantibodies in the thyroid and different degrees of thyroid follicle destruction, eventually leading to hypothyroidism [5]. The prevalence of HT is 2% in the general population, which continues to rise; females are significantly more likely to have HT [6]. Current studies indicate that there is a correlation between the occurrence of HT and OLP. This article reviews the potential mechanisms involved in the cooccurrence of these diseases.

## 2. Clinical Studies of the Cooccurrence of OLP and HT

The correlation between OLP and thyroid disease was first reported in 1994 [7]. Robledo-Sierra et al. [8] found that OLP lesions in patients with concomitant thyroid disease presented differently over time, indicating a specific OLP subgroup. Based on previously published reports [9], hypothyroidism and Hashimoto's thyroiditis are the thyroid diseases most commonly associated with OLP. Amato-Cuartas et al. [10] found that the prevalence of hypothyroidism in Colombian patients with OLP was 35.7%, compared with 3.95% in the entire study population. A number of studies have also examined the relationship between OLP and HT. Lo et al. [11] found that the prevalence of HT among OLP patients was 14.3%, whereas the prevalence of HT in the general population was 2% [6]; the authors suspected that HT plays a causal or predisposing role in OLP. A case-control study in China also suggested that there is a close relationship between OLP and HT [12]. In another study, Li et al. [9] combined data from four articles published between 2010 and 2016 and conducted a meta-analysis. They concluded that there was a significant association between OLP and HT, suggesting that these two diseases share a common pathogenesis.

## 3. Potential Mechanisms Underlying the Cooccurrence of OLP and HT

### 3.1. Immune Factors

The histopathological features of OLP and HT indicate that the cell-mediated immune response plays an important role in their pathogenesis [13, 14]. The typical histological features of OLP include subepithelial band-like infiltration of lymphocytes (mostly T lymphocytes), liquefaction degeneration of basal epithelial cells, and hyperparakeratosis. These features may be related to basal keratinocyte damage caused by CD4+ T cell activation by antigen-presenting cells or CD8+ T cell activation by basal keratinocytes [15]. HT is characterized by lymphocyte infiltration and thyroid fibrosis. Collectively, both OLP and HT involve inflammatory infiltration, predominantly containing T cells. Therefore, the occurrence of both diseases involves immune-related pathological processes. This suggests a potential immune mechanism underlying the cooccurrence of these two diseases.

#### 3.1.1. Thyroid-Specific Antibodies

In HT, cell- and antibody-mediated humoral immune responses against thyroid gland self-antigens cause thyrocyte destruction, subsequently resulting in hypothyroidism. Human thyroid autoantibodies include thyrotropin receptor antibody (TRAb), thyroglobulin antibody (TGAb), thyroid peroxidase antibody (TPOAb), and anti-sodium iodide symporter (NIS) antibodies. TGAb and TPOAb are present in nearly all HT patients. TPOAb are the best serological marker for diagnosing HT, occurring in approximately 95% of HT patients, while TGAb are less sensitive and less specific [16].

In the past decades, many studies have reported a significant link between thyroid diseases, especially autoimmune thyroid diseases (AITD) and autoimmune skin disease [17]. In AITD patients, the skin is targeted by autoantibodies against thyroid-specific antigens [18] and the prevalence of skin diseases among thyroid disease patients is very high [19]. TPOAb and TGAb can induce epithelial cell damage [20]. Keratinocytes, which express thyroid-stimulating hormone receptor (TSHR) and TG, can be recognized and targeted by TRAb and TGAb in HT patients [21]. Keratinocytes do not express TPO; however, given that TPOAb are of greater pathogenetic importance than TGAb in HT, many researchers have hypothesized that circulating TPOAb may cross-react with unknown proteins on keratinocyte membranes [11]. Once bound to the targets on the keratinocyte surface, thyroid autoantibodies may trigger CD95- (Fas/Apo-1) mediated apoptosis [22]. Apoptotic bodies may then be internalized and processed by surrounding keratinocytes or antigen-presenting cells, leading to T cell activation. Basal keratinocytes are then targeted by cytotoxic T cells, eventually leading to the occurrence of OLP. Although the precise mechanisms for autoantibody induction and production in the sera of OLP patients are unknown, AITD may lead to the production of antigens in the damaged thyroid tissue, which then activate antigen-specific B cells to produce antibodies locally and in the blood circulation of OLP patients. The severity of OLP lesions is directly linked to TPOAb levels [23]. In addition, circulating thyroid antibodies in HT patients contribute to the triggering of oral mucosa-specific autoimmune responses, leading to OLP [11]. As oral mucosal keratinocytes can express TSHR and TG, which can be recognized by TRAb and TGAb, we hypothesize that HT patients may have secondary OLP damage. In conclusion, the coexistence of OLP and HT is related to TG expression in oral keratinocytes.

#### 3.1.2. CD8+ T Cells

CD8+ T cells are an important branch of the adaptive immune system; they contribute to the clearance of intracellular pathogens and provide long-term protection [24]. These functions are mostly fulfilled by the best-characterized CD8+ T cell subpopulation, the cytotoxic T lymphocytes, owing to their capacity to kill infected cells and secrete cytokines such as interferon- (IFN-) *γ* and tumor necrosis factor- (TNF-) *α* [25].

Both antigen-specific and nonspecific mechanisms may be involved in OLP pathogenesis. A key element of pathogenesis is that T lymphocyte-mediated cytotoxicity leads to keratinocyte apoptosis. Most T cells adjacent to damaged basal keratinocytes are CD8+ T cells. CD8+ cytotoxic T cells can be directly activated by antigens binding to major histocompatibility complex- (MHC-) 1 on keratinocytes and subsequently release chemokines that attract additional lymphocytes and other immune cells into the developing OLP lesion [26]. HT pathogenesis involves perforin and granzyme release by CD8+ cytotoxic T cells, resulting in thyroid cell damage and eventually leading to hypothyroidism [27]. Additionally, approximately 2–3% of infiltrating CD8+ cells can recognize TPO/TG and function in thyroid tissue destruction, thus leading to clinical disease [28].

#### 3.1.3. CD4+ T Cells

CD4+ T helper (Th) cells can differentiate into several distinct subsets, including Th1, Th2, Treg, Th17, Th22, and Tfh, which produce specific cytokines [29]. Th1 cells mainly produce IFN-*γ*, TNF-*α*, and interleukin- (IL-) 2, which mediate cellular immune responses. Th2 cells predominantly produce cytokines, such as IL-4, IL-5, IL-10, and IL-13, which mediate humoral immune responses. The IFN-*γ*/IL-4 ratio is a simple and direct indicator of the Th1/Th2 balance. Previous studies have shown that both IFN-*γ* and IL-4 levels are increased in the serum and lesion tissues of OLP patients compared with healthy controls and that the IFN-*γ*/IL-4 ratio is also significantly increased, indicating that OLP is Th1-biased [30]. Other studies have shown that the TPOAb titer is correlated with increased Th1 cytokine production in HT patients [31]. HT patients have higher IFN-*γ* and lower IL-4 serum levels, further indicating the Th1 bias in HT [32]. Collectively, these results demonstrate that both OLP and HT are predominantly Th1-type cytokine diseases.

Th17 cells are an independent subset of helper T cells distinct from the development of Th1 and Th2 cells. Th17 cells can produce IL-17 and play a critical role in autoimmune diseases. IL-17 can induce keratinocytes, fibroblasts, endothelial cells, and macrophages to secrete TNF-*α* and IL-2 and subsequently promote inflammation [33]. Furthermore, the proportion of peripheral Th17 cells and IL-17 serum levels is significantly higher in OLP patients than controls, indicating that Th17 cells are involved in OLP immunopathogenesis [34]. Correspondingly, Th17 cells and serum IL-17 levels are significantly higher in HT patients [35]. IL-17 can promote inflammatory mediator secretion and T cell proliferation, leading to thyroid tissue inflammation, thyroid autoantibody production, and secondary tissue damage.

Th22 cells are a novel CD4+ T subset that mainly secretes IL-22, which is associated with many autoimmune diseases such as systemic lupus erythematosus and rheumatoid arthritis [36]. There is no direct evidence showing an increase in Th22 cells in OLP lesions. However, IL-22 levels in OLP patients are significantly higher than in controls [37]. IL-22 promotes keratinocyte proliferation and epithelial hyperplasia; therefore, IL-22 may promote epithelial hyperplasia in local OLP lesions [38]. Previous studies have shown that IL-22 levels in the peripheral blood of HT patients are significantly higher than in controls, while Th22 cell levels were positively correlated with the TPO antibody titer, suggesting that Th22 cells may be involved in HT pathogenesis [39].

The regulatory T (Treg) cell subset produces cytokines, including transforming growth factor- (TGF-) *β* and IL-10, via direct or indirect cell contact, and subsequently inhibits the immune response [40]. The main surface markers of Treg cells are CD25 and Foxp3. In most autoimmune diseases, such as type I diabetes mellitus, rheumatoid arthritis, and primary Sjogren's syndrome, a decrease in Treg cell levels or dysfunction can be detected [41, 42]. One study showed functional deficiency of Treg in HT patients, suggesting that the role of Treg cells in HT is consistent with their role in other autoimmune diseases [43]. Accordingly, the proportion of CD4+CD25+Foxp3+ Treg cells in the peripheral blood is significantly higher in OLP patients than in the controls and these cells mainly infiltrate epithelial and superficial connective tissue, which is adjacent to basal keratinocytes [44]. This suggests that Foxp3+ Treg cells play a role in OLP pathogenesis, which may be related to T cell resistance caused by decreased sensitivity of effector CD8+ T cells to Treg during OLP pathogenesis. To maintain immune homeostasis, Treg cells exert their compensatory ability and subsequently enhance immune suppression [45]. However, the Th17/Treg cell ratio in the peripheral blood of both OLP [46] and HT [47] patients is increased. As Th17/Treg axis disorders (especially the reduction, or relative reduction, in Treg cell levels and increase in Th17 cell activity) are involved in the pathogenesis of many autoimmune diseases [48], we hypothesize that imbalance of the Th17/Treg axis may underlie the occurrence and cooccurrence of OLP and HT.

Follicular helper T (Tfh) cells are a newly identified T helper cell subset, which can promote the generation of antigen-specific B cells by producing IL-21 [49]; these cells also express the chemokine receptor CXCR5 and inducible costimulatory (ICOS) protein. Previous studies have shown that the number of Tfh cells is increased in the peripheral blood of HT patients, which correlates with thyroid-specific antibody levels [50]. A more recent study found that the peripheral blood of OLP patients has significantly increased CXCR5+CD4+ Tfh-like cell and B cell levels, along with significantly reduced serum IL-21 levels, suggesting that increased circulating Tfh-like cells may participate in OLP pathogenesis via the abnormal regulation of B cell proliferation and IL-21 production [51].

#### 3.1.4. Chemokines

Chemokines are proinflammatory cytokines, which are classified into C, CC, CXC, and CX3C subfamilies according to their N-terminal cysteine motifs. Chemokines play a key role in the selective recruitment of T cells via chemokine receptors [52]. CXCL10, an IFN-*γ*-induced chemokine, participates in the pathogenesis of many autoimmune diseases by binding to CXCR3 [53]. CXCL10 is secreted by many cell types including endothelial cells, fibroblasts, keratinocytes, thyroid cells, and preadipocytes. A high level of circulating CXCL10 is an efficient marker of the host immune response, especially the Th1 immune response [54]. Previous studies have shown that CXCL10 expression in the epithelial layer of OLP lesions is significantly higher than in normal tissues [55]; furthermore, real-time quantitative PCR analysis of lamina propria samples from OLP patients demonstrated enhanced expression of CXCR3. CXCL10 expression is also increased in the serum and tissues of AITD patients [56]. Several current ongoing studies are focused on exploring innovative HT therapies by developing and evaluating new molecules that can antagonize CXCR3 or block CXCL10 [57]. Another chemokine, RANTES, a member of the CC chemokine family, regulates the activation, expression, and secretion of T cells [58]. RANTES plays a crucial role in the recruitment of lymphocytes, monocytes, natural killer cells, eosinophils, basophils, and mast cells in OLP [59]. RANTES serum levels are significantly higher in HT patients than in controls [60], suggesting that RANTES may be involved in HT pathogenesis.

**Figure 1:**
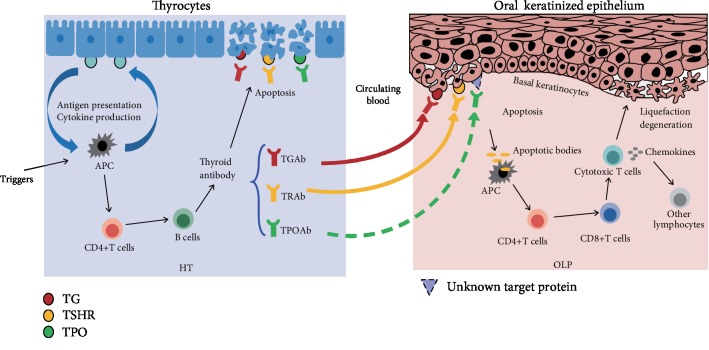
Basal keratinocytes can express TSHR and TG, which can be recognized by TRAb and TGAb in HT patients; circulating TPOAb may cross-react with unknown proteins on keratinocyte membranes. Once bound to the targets on the keratinocyte surface, thyroid autoantibodies may trigger apoptosis of basal keratinocytes. Antigen-presenting cells phagocytose the apoptotic bodies from basal keratinocytes and present them to T helper cells, which in turn stimulate cytotoxic T cells against basal keratinocytes. Activated CD8+ cytotoxic T cells can release chemokines that attract additional lymphocytes and other immune cells into the developing OLP lesion.

In summary, keratinocyte expression of TSHR and TG, which can be recognized and targeted by TRAb and TGAb in HT patients, is the basis for the cooccurrence of OLP and HT (Figure 1). In addition, the important role of TPOAb, CD8+ T cells, CD4+ T cells, and chemokines in the pathogenesis of these two diseases suggests that other possible immune mechanisms are involved in the pathogenesis of these two diseases.

### 3.2. Environmental Factors

Environmental factors play an important role in the pathogenesis of OLP and HT. However, the relationship between smoking and OLP remains unclear. Although some studies have indicated that smoking is unrelated to OLP [61, 62], Neumann-Jensen et al. [63] highlighted that OLP is less common among smokers than nonsmokers. The relationship between smoking and HT is also controversial. A population-based case-control study showed that smoking cessation is followed by a sharp but transient rise in the incidence of HT [64]. A study involving individuals with genetic predisposition to HT showed that fewer patients progressed to significant hypothyroidism in the smoking group than in the nonsmoking group [65]. A recent study has suggested that smoking confers a protective effect for OLP and thyroid gland diseases, which may affect the establishment of a possible link between OLP and HT [66].

Infectious factors can also result in immune abnormalities [67]. Hepatitis C virus [68, 69] and human herpesvirus 6 [70, 71] are associated with both OLP and HT, while other studies have reported conflicting results regarding the association of Epstein-Barr virus [72, 73] and *Helicobacter pylori* [74, 75] with these diseases.

Vitamin D has a beneficial effect on the immune system, and inadequate vitamin D intake is involved in many autoimmune diseases [76]. Macrophages, dendritic cells, monocytes, T lymphocytes, and B lymphocytes all express the vitamin D-activating enzyme CYP27B1 and the vitamin D receptor (VDR) [77–79]. Active vitamin D, 1,25(OH)_2_D, derived from 25(OH)D, binds to VDR and regulates the proliferation and differentiation of immune cells, leading to reduced lymphocyte activity (especially in Th1-type lymphocytes), and reduces proinflammatory cytokine expression [80]. Previous studies have shown a lack of VDR in OLP lesions and reduced 1,25(OH)_2_D and 25(OH)D serum levels compared to levels in the controls [81]. Current evidence suggests that vitamin D deficiency is associated with thyroid autoimmunity, which may play an important role in HT immunopathogenesis [82]. Lack of vitamin D in patients with OLP, HT, and OLP with HT may lead to enhanced Th1 lymphocyte activity, which promoted inflammatory cytokine expression, and finally disease onset.

Many animal and human studies have shown that stress can induce a variety of immunological changes that are associated with several autoimmune diseases [83–85]. Recent studies have shown that stress can affect the immune system via direct or indirect neuromodulation and endocrine regulation and subsequently affects the Th1/Th2 balance [86]. One American study has shown that OLP is closely linked to a tense mental state [87]. More recent studies have also shown that anxiety, depression, and psychological stress are closely correlated with OLP incidence, indicating that psychosocial stress is an important cause of OLP [88]. While mental and psychological factors are both associated with AITD development, few studies have investigated their effects on HT, mostly because the pathogenesis and course of HT are generally recessive [89]. However, the change in immune response balance caused by psychosocial factors is probably one of the mechanisms underlying OLP and HT and their cooccurrence.

### 3.3. Hormones

The role of estrogen and progesterone (Pg) in autoimmune disorders, such as multiple sclerosis, systemic lupus erythematosus, and rheumatoid arthritis, has been established [90, 91]. Epidemiological studies have revealed significant gender differences in the incidence of OLP and HT; the ratio of males to females is approximately 1 : 1.4 for OLP [26] and 1 : 5 to 1 : 10 for HT [6]. The ratio of males to females with OLP complicated by HT is approximately 1 : 12 in China. These gender differences suggest that sex steroid hormones probably play a significant role in the pathogenesis of OLP and HT. Although estrogen can enhance humoral immunity, its effect on cellular immunity remains controversial. Estrogen regulates all T cell subsets including CD4+ T cells (Th1, Th2, Th17, and Treg) and CD8+ T cells. Changes in estrogen levels can interfere with autoimmunity [92]. Although no direct relationship has been established to date between estrogen and OLP, OLP is more common in perimenopausal women [93], which may be related to hormonal fluctuations during menopause. Regarding HT, it has been confirmed that estrogen is closely involved in AITD and estrogen receptor expression is heightened in HT patients [94]. These results suggest that estrogen is closely associated with the pathogenesis of OLP and HT and that it may influence their cooccurrence.

### 3.4. Hereditary Factors

Human leukocyte antigen (HLA) is the main histocompatibility complex in humans; HLA polymorphism is a key genetic factor underlying many diseases [95]. HLA-encoded molecules are expressed on the surface of different cells and participate in antigen presentation, restrict cell recognition, and induce immune responses; consequently, nearly all autoimmune diseases are related to HLA. Studies examining the association between HLA alleles in HT and OLP have demonstrated a common genetic susceptibility for these two diseases [96]. The HLA-DRw9 allele is closely related to autoimmune diseases, including HT [97]; this allele is highly prevalent in Chinese OLP patients [98]. Other studies have shown that HT is associated with HLA-DR3 alleles in Caucasians [99] and that HLA-DR3 is closely related to the erosive variant of OLP [100]. These studies suggest that OLP and HT may have the same genetic background and that these genetic factors are directly involved in the abovementioned immune responses.

## 4. Conclusions

**Figure 2:**
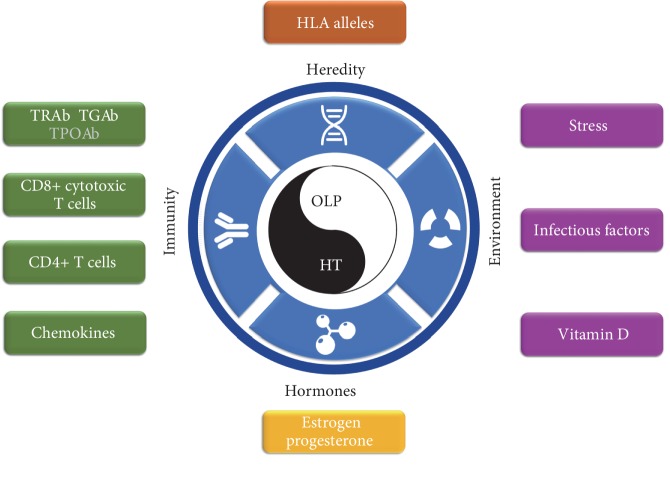
Current evidence regarding potential mechanisms underlying the co-occurrence of OLP and HT.

This review summarizes and discusses the mechanisms underlying the cooccurrence of these two diseases, which might be related to a range of immune, environmental, endocrine, and genetic factors (Figure 2). These factors can eventually lead to the occurrence or cooccurrence of OLP and HT via various mechanisms that ultimately affect the Th1/Th2 balance. In addition, we hypothesize that OLP might be secondary to HT in some cases of OLP and HT cooccurrence. Although the specific processes and mechanisms involved in this cooccurrence need to be further investigated, this review poses a novel strategy: a routine screen for thyroid diseases, particularly HT, is recommended upon the initial diagnosis of OLP. This procedure will facilitate early HT diagnosis and treatment.
